# Analysis of 19 Minerals and Cortisol in Red Deer Hair in Two Different Areas of the Stelvio National Park: A Preliminary Study

**DOI:** 10.3390/ani9080492

**Published:** 2019-07-26

**Authors:** Marta Montillo, Chiara Caslini, Tanja Peric, Alberto Prandi, Paola Netto, Franco Tubaro, Luca Pedrotti, Alessandro Bianchi, Silvana Mattiello

**Affiliations:** 1 Dipartimento di Scienze Agroalimentari, Ambientali e Animali, DI4A, Università degli Studi di Udine, 33100 Udine, Italy; 2 Dipartimento di Medicina Veterinaria, Università degli Studi di Milano, 20133 Milano, Italy; 3 Parco Nazionale dello Stelvio, 23032 Bormio (SO), Italy; 4 Istituto Zooprofilattico Sperimentale della Lombardia e dell’Emilia-Romagna “Bruno Ubertini”—Sezione di Sondrio, 23100 Sondrio, Italy

**Keywords:** hair, deer, mineral, cortisol

## Abstract

**Simple Summary:**

Minerals play an important role in animals’ health and nutritional status and are associated with cortisol, a hormone involved in several physiological processes and with a fundamental role in allostasis, the process of achieving homeostasis. The aim of the present study is to perform a preliminary investigation on the concentration of 19 minerals and cortisol in the hair of red deer coming from two different areas of the Stelvio National Park. Results showed a close association between hair minerals and cortisol concentrations and an effect of deer origin. Hair minerals and cortisol assessment is an easy, rapid, and low cost screening method and it is useful in wildlife management programs, in order to investigate wild animals’ health status, to perform environmental studies, to assess the presence of contaminants in wild species and to determine risks for humans. In addition, it could be helpful in public health programs to estimate contamination risks linked to wild animal meat consumption or to assess the presence of contaminants in food-producing animals.

**Abstract:**

The aim of the study was to perform an investigation on the concentration of 19 minerals and cortisol in red deer (*Cervus elaphus*) hair, a matrix that is easy to collect with non-invasive and painless sampling, able to represent an integrative values of long-term substance concentrations, and able to give useful information, also when performed on dead animals, given its extreme stability over time. In the study thirty-five animals were included, coming from two different sides of a valley in the Stelvio National Park, where official water analysis had pointed out elevated concentrations of As in one of the two orographic sides. Hair cortisol concentrations were measured using a RIA(Radio Immuno Assay), while minerals were detected using ICP-MS (Inductively Coupled Plasma- Mass Spectrometry). Results showed a negative relationship between cortisol and some mineral concentrations (Li, Co, As, Cd, Cr and Tl) and significant differences in some mineral concentrations between park areas (Al, Co, Cu, Cd and Ni). As, Cr and cortisol differences approached statistical significance. This preliminary study represents a step forward in the study of wildlife allostatic load and a valid method for applications in wildlife management programs, in environmental studies and in public health programs.

## 1. Introduction

Minerals play an important role in animals’ health and nutritional status. Some of them (such as selenium, zinc and copper) may positively preserve animal immune functions [1], fertility [2] and weight gain [3,4]. Magnesium is the main intracellular divalent cation and it is essential for several enzymatic functions [5,6,7,8]. Phosphorus is implicated in energy metabolism, intracellular signaling, nucleic acid synthesis and cell structure [5,6,7,8]. Zinc is involved in the synthesis and degradation of carbohydrates, lipids, proteins and nucleic acids and it is involved in a large number of enzymes implicated in gene transcription [5,6,7,8].

Several authors reported the associations between deficiencies in mineral content with disease syndromes, lack of resistance, and poor reproductive performance in black-tailed deer (*Odocoileus hemionus columbianus*), elk (*Cervus canadensis*), and moose (*Alces alces*) populations [9,10,11,12,13,14,15]. On the other hand, some heavy metals, such as cadmium (Cd), lead (Pb), mercury (Hg) and arsenic (As), can be toxic even at low concentrations, and their content may show a great variability depending on several factors [16]. In particular, the chronic form of As toxicity in animals includes particular fibrosis producing stiffness and asymmetrical enlargement of hocks or other joints of the limbs, in coordination with ataxia and posterior paresis [17]. In general, hair mineral content can reflect soil differences, diet selection, climate-controlled mineral levels in foraging and proximity to pollution sources [18,19,20]. Mineral concentration has also been found to be closely associated with stress conditions, health status, resistance of animals [21], and cortisol levels [22,23,24]. In fact, the production of stress hormones, such as cortisol, shifts the body’s metabolism to a catabolic state, thereby increasing oxidative stress and increasing the need for anti-oxidants (e.g., minerals for enzyme function) [25,26]. Cortisol is a steroid hormone involved in several physiological processes, such as the stress response, the regulation of blood sugar through gluconeogenesis, the suppression of the immune system, and the metabolism of fat, proteins and carbohydrates [27]. Cortisol plays a fundamental role in allostasis, the active process of maintaining and/or reestablishing homeostasis that helps an animal to adapt to a new situation and/or challenge [28]. High concentrations of this steroid over long periods can result in a pathological syndrome featuring metabolic changes and the depression of reproductive and immune functions, with direct effects on the central nervous system [29,30,31]. Moreover, in humans, stress has been associated with common gastro-intestinal disorders and the inhibition of nervus vagus activation, perturbing gastric emptying, gastroduodenal/colonic motility and intestinal transit [32,33,34]. Stress hormones may also directly affect mineral absorption, distribution or excretion (e.g., cortisol influences the parathyroid hormone and renal calcium handling, and therefore affects calcium homeostasis) [35]. In addition, increased competition among free-ranging animals could imply an increase in hair cortisol concentrations, but may also imply a decrease in food intake and in turn a decrease in mineral absorption.

Thus, the measurement of mineral and cortisol concentrations is important for monitoring the animals’ adaptation to the environment in which they live and in the last decades, the hair matrix has been increasingly used in the analysis of mineral and steroid concentrations. This matrix provides a “retrospective picture” of previous minerals and hormones accumulation and incorporation from plasma over a period of time [36,37,38,39,40,41]. Elements linked to the hair shaft are unaffected by circadian hormone variations or by factors that induce short-term variations. Hair provides an integrated rather than a one-time point measure [42], with no need for repeated sampling of individuals. Moreover, hair matrix is easy to collect in live animals, with its non-invasive and painless sampling. It also gives useful information, also when performed on dead animals, given its extreme stability over time [43].

Some studies used hair for mineral detection in ecological and clinical investigations [44,45,46,47] and the Global Environmental Monitoring System (GEMS) of the United Nations Environmental Program chose hair as the biological matrix for the biological monitoring of minerals [48].

Several studies on environmental contamination were performed using hair from wild animals. Levels of heavy metals were determined in the Alaska moose (*Alces alces gigas*) [49], reindeer (*Rangifer tarandus*) [50], brown bear (*Ursus arctos*) [31], wild boar (*Sus scrofa*) [51], squirrel (*Sciurus vulgaris*) [52], opossum (*Didelphis virginiana*) in Costa Rica [53] and in wild boar in Central Italy [54]. Hair mineral content was also evaluated in several deer species for different purposes, such as the estimation of soil productivity in white-tailed deer (*Odocoileus virginianus*) [18,19], the assessment of individual or population health in mule deer (*Odocoileus hemionus*) [15] and red deer [55], or for environmental studies in roe deer (*Capreolus capreolus*) [56] and moose (*Alces alces*) [14]. Hair steroid analysis has been largely used in wildlife [57,58,59], but also for the detection of steroid concentrations in many studies to evaluate the stress response, consequently to evaluate the animal allostatic load [36,60], environmental conditions [36] and the adaptation to environmental or physiological changes [61,62,63,64]. Given the importance of the study of minerals and cortisol as an important mechanism linking ecological change with impaired wildlife population health, and given the potential of hair analysis for obtaining useful information from free-ranging wild animals, the aim of the present study was to perform a preliminary investigation about the concentration of 19 minerals (Li, B, Al, Ti, V, Cr, Fe, Ni, Co, Cu, Zn, As, Ag, Cd, Sn, Sb, Ba, Tl and Pb) and cortisol in the hair of red deer coming from two different sides of an alpine valley in the Stelvio National Park, where official water analysis had pointed out elevated concentrations of As in the left orographic side [65].

## 2. Materials and Methods

### 2.1. Ethics

All applicable international, national, and/or institutional guidelines for the care and use of animals were followed.

Samples were collected from red deer culled during an intervention of biological control (January and February 2012), in the frame of the revised version of the “Plan for conservation and management of red deer in the Lombardy sector of the Stelvio National Park”, which received a positive judgment by the National Higher Institute for Environmental Protection and Research (I.S.P.R.A.) and was approved in June 2010 by the Italian Ministry of the Environment.

### 2.2. Study Area

**Figure 1:**
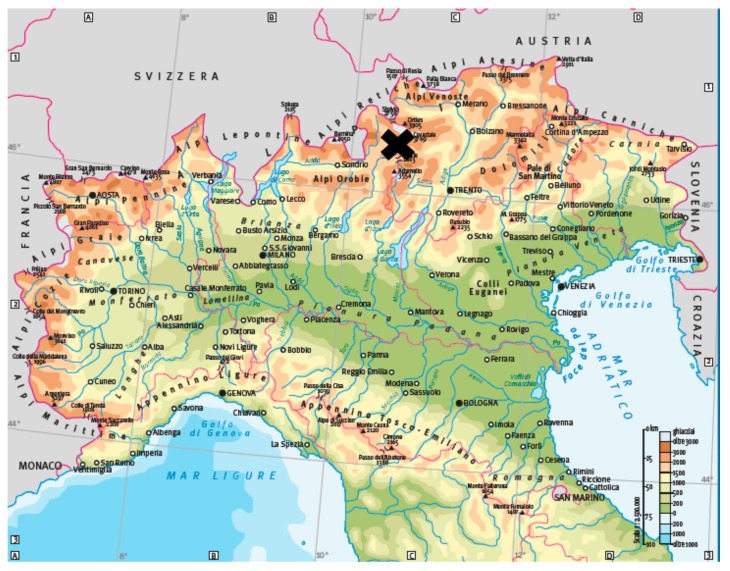
Position of Valfurva with respect to Italy.

The present study was performed in Valfurva, an alpine valley in the Central Italian Alps, within the Lombard sector of the Stelvio National Park (SNP) (Figure 1).

The SNP presently hosts a numerically important red deer population, with a deer density of up to 31 deer/km^2^ in winter areas (density in actually occupied areas), which exceeds the local carrying capacity with serious impacts on agriculture, forestry and road killings; for this reason. The pre-reproductive red deer density in Valfurva during the study period was around 11 deer/km^2^ [66].

In order to reduce the density of red deer in the SNP and to control excessive red deer impact on agriculture, forestry and road killings, an authorized culling program was started in 2011, in the frame of the national law n. 394/1992.

We assumed that during the control period individuals were rather stable in the area where they were culled. This assumption was based on the results of the analysis of spatial behavior during winter time of about 100 red deer that were monitored by radio-tracking for some years in SNP. These results allowed to define some sub-populations within the SNP territory and showed that after the rut most red deer stags reach their winter districts (when they differed from their rut districts) and they establish themselves there, until the subsequent spring. In this phase, they are highly sedentary and just some individuals move to different areas [67].

From a geological point of view, in the Lombard sector of the SNP we can identify two large units, characterized by a clearly differentiated geological substrate, which consequently also influenced the composition of soil and vegetation. The North-West (NW) area consists of stratified sedimentary rocks of calcareous-dolomitic origin (dolomite, dolomitic limestone and marly limestone) dating back to the Mesozoic period. The presence of a lithological substratum of calcareous origin has given rise to superficial soils, which are generally dry and of poor fertility. In this area, we can commonly observe formations dominated by *Pinus mugo* and pastures mainly dominated by *Sesleria coerulea* that are scarcely palatable and not very productive. The South-East (SE) area, which represents about 4/5 of the whole Lombardy sector, is composed of metamorphic rocks of schist, mainly quartziferous phyllites, paragneisses, gneisses and mica schists [67,68]. These geo-lithological formations gave origin to soils with good pedological characteristics, of average depth, suitable for the development of forest vegetation. The presence of a siliceous substrate favored the formation of woods dominated by *Picea abies* in the mountain and subalpine planes. *Pinus cembra* is also widely present in the subalpine plane, whereas *Alnus viridis* can be frequently found in the proximity of streams and gullies on the north-facing slopes. At the level of alpine meadows, the most frequent formations are highly productive and highly palatable meadows dominated by *Carex curvula* and *Festuca spp*. [67,69]. In this SE area, particularly in the Valfurva township, As concentrations exceeded the acceptable thresholds established by the Italian law (<50 μg/L, value prescribed by D. Lgs. no. 152/2006), as reported by the National Agency for Environmental Research and Protection [65,70]. On the other hand, the NW area didn’t show any exceeding of the threshold values.

**Figure 2:**
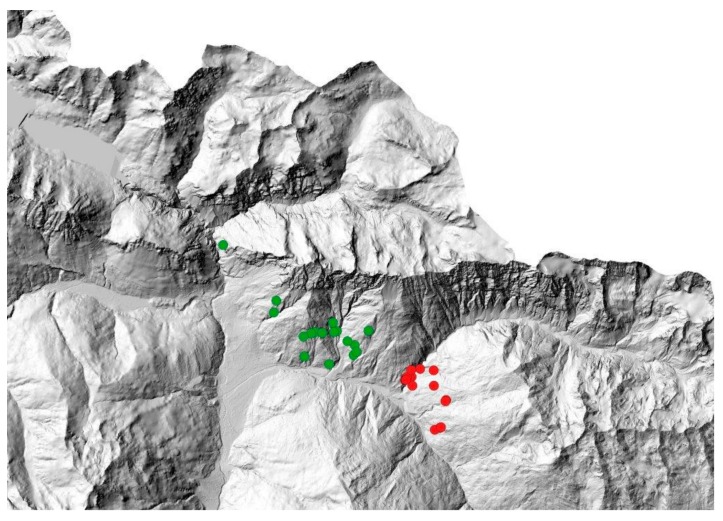
Localization of red deer cullings in Valfurva. Red circles represent individuals culled in the South-East (SE) area; green circles represent individuals culled in the North-West (NW) area.

Given the different characteristics of the two areas of the Lombardy sector, samples were classed depending on the culling site as NW and SE (Figure 2). Deer density in the winter areas did not significantly differ between the NW and SE culling sites [67].

### 2.3. Hair Sampling and Data Collection

Thirty-five red deer were culled in Valfurva (18 in NE and 17 in SW) and individually identified by a numerical code. Sex, age, and biometric measures were recorded in a dataset containing also the geographic coordinates of the culling site and the date of culling. Animals were classed depending on their age as: adults (i.e., >2 years old; *n* = 13; 5 females and 8 males), yearlings (1–2 years old; *n* = 6, 1 female and 5 males), and calves (<1 year old; *n* = 16; 10 females and 6 males).

Biometric measures were collected following the guidelines by Mattioli and De Marinis [71] and included: body length (cm), foot length (cm), height at withers (cm), jaw length (mm), and carcass weight of completely eviscerated and not skinned animals (kg). Furthermore, the Kidney Fat Index (KFI) was calculated as a measure of nutritional status and expressed as the percentage of the total weight of fat around the kidneys out of kidney weight [72].

A patch of hair was collected from the lower back region of each deer. Electronic clippers were used to shave the hair close to the skin. Samples were dried from water if necessary under a gentle air stream and stored in paper envelopes in the dark and at room temperature until being analyzed.

### 2.4. Hair Washing Procedure

Three different rations of 250 mg of hair samples were placed in polypropylene tubes, covered with isopropanol (Merck KGaA, Darmstadt, Germany) (5 mL) and gently mixed for 3 min at room temperature. The sample was again washed with isopropanol and air dried. This washing procedure minimize the risk of extracting cortisol and minerals from outside the hair and also to ensure the removal of any steroids on the surface of the hair due to sweat and sebum. Subsequently, hair were mixed uniformly and divided in two portions for minerals and cortisol analysis. Hair samples were approximately 5 cm long.

### 2.5. Hair Minerals Assay

Approximately 0.5 g of the sample was digested with 2.5 mL of HNO_3_ (65%) (Merck KGaA, Darmstadt, Germany) and 0.5 mL H_2_O_2_ (Merck KGaA, Darmstadt, Germany) in a microwave digestion system (Milestone Inc., Shelton, CT, USA). The temperature program was as follows: 2 min at 250 W, 2 min at 0 W, 5 min at 250 W, 8 min at 500 W and 5 min 750 W. The resulting solutions were cooled and diluted to 100 mL with deionized water. The entire procedure was checked for accuracy using three independent measurements. Each analytical run also included standard reference material with known concentrations of each mineral were carried out by using the same procedures. The clear solutions were analyzed by NexION 350× ICP-MS (Inductively Coupled Plasma- Mass Spectrometry, PerkinElmer Life Sciences Inc., Boston, MA, USA) with a pneumatic nebulizer. For calibration, standard solutions were prepared from the stock standard solution of 1000 ng/mL by dilution. The ranges of the calibration curves (six points) were selected to match the expected concentrations for the element of the sample investigated by ICP-MS. Linearity was checked in the range of 0–1000 μg/g. Detection limits were calculated as the concentrations of an element that gave a signal equal to three times the standard deviation of a series of five successive measurements of the blank solution at the element peak.

### 2.6. Hair Cortisol Assay

Hair cortisol assay was performed as explained in Caslini et al. [36]. Hair cortisol was extracted with 3 mL of methanol (Merck KGaA, Darmstadt, Germany) per 40 mg of hair for 18 h at 37 °C. Samples were then centrifuged (15 min/200 g) and the supernatant collected and transferred to a 12-mm glass test tube. The supernatant was dried at 37 °C under a gentle stream of nitrogen gas and reconstituted with 0.3 mL of phosphate buffer. Hair cortisol concentrations were measured using a solid-phase microtiter RIA (Radio Immuno Assay). In brief, a 96-well microtitre plate (OptiPlate, PerkinElmer Life Sciences Inc., Boston, MA, USA) was coated with goat anti-rabbit γ-globulin serum (Analytical Antibodies, Bologna, Italy), (diluted 1:1000 in 0.15 mM sodium acetate buffer at pH 9) and incubated overnight at 4 °C. The plate was then washed twice with RIA buffer (pH 7.4) and incubated overnight at 4 °C with 200 μL of the anti-cortisol serum diluted 1:12,000. The rabbit anti-cortisol antibody was obtained from Biogenesis (Poole, UK). After washing the plate with RIA buffer, standards (5–300 pg/well), a quality control extract, the test extracts (10 mg of hair), and tracer (Hydrocortisone (Cortisol, [1,2,6,7-3H (N)]-), PerkinElmer Life Sciences Inc., Boston, MA, USA) were added, and the plate was incubated overnight at 4 °C. Bound hormone was separated from free hormone by decanting and washing the wells in RIA buffer. After the addition of 200 μL/well scintillation cocktail (Microscint 20, PerkinElmer Life Sciences Inc., Boston, MA, USA), the plate was counted using a beta-counter (Top-Count, PerkinElmer Life Sciences Inc., Boston, MA, USA).

The assay sensitivity (defined as the hormone concentration producing a displacement of the labeled hormone at least two standard deviations from maximal binding) was 1.23 pg/well. The specificity of the method, estimated by calculating the percentage cross-reaction with different steroids, was: cortisol 100%, corticosterone 1.8%, and aldosterone <0.02%. The precision of the method was estimated by repeatedly assaying samples in the same assay and in independent assays was expressed by intra-assay and inter-assay coefficients of variation (CV%) of the hair sample. The intra- and inter-assay coefficients of variation were 3.6% and 9.8%, respectively. Assay validation has been performed in Caslini et al. [36].

### 2.7. Statistical Analysis

Results of some samples were not considered for statistical analysis, due to fact that mineral concentrations were undetectable (lower than standard curve). Therefore, sample size is not always the same for all minerals. Details on sample size are reported in Table 1.

Preliminary analysis pointed out the presence of four individuals (two young adult stags, one adult hind and one calf; three in the NW, and one in the SE area) whose mineral concentrations fell completely out of the distribution of other samples from the same culling area (NW or SE) for more than 25% of the minerals, showing values outside an interval of three units of standard deviations around the mean; these individuals were considered as outliers, and were therefore removed from the dataset.

Preliminary analysis also confirmed the lack of any effect of sex and age class on hair mineral concentration, in agreement with previous studies on red deer [55]. Therefore, for subsequent analyses, samples were classed only depending on the culling area, disregarding the effect of sex and age class.

Data exploration was carried out using principal component analysis (PCA; IBM Corp. Released 2017. IBM SPSS Statistics for Windows, Version 25.0. Armonk, NY: IBM Corp.) on mineral and cortisol concentrations. Unfortunately, due the presence of missing values, Ag, B, Cd, Pb, Sb and Sn could not be included as variables for this analysis.

As cortisol and mineral concentration data were not normally distributed, the differences in cortisol and all analyzed minerals concentrations between the NW and SE samples were tested using non parametric analysis of variance (Mann–Whitney test), whereas biometric data were normally distributed and were therefore analyzed within each age class (calves, yearlings and adults) using the *t*-test (IBM SPSS Statistics, Vers. 25).

Spearman correlation ranks were calculated between cortisol and mineral concentrations (IBM SPSS Statistics, Vers. 25).

## 3. Results

**Figure 3:**
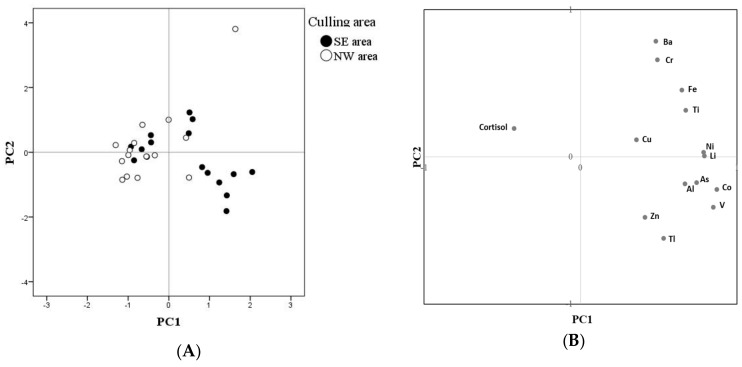
Principal component analysis (PCA) results on the first two principal components (PC1: 41.63% of explained variance; PC2: 15.13% of explained variance). (**A**) Score plot, representing the location on the first two principal components of each sample, classed depending on the culling area: South-East (SE) or North-West (NW); (**B**) loadings plot, representing the weight of each variable on the first two principal components.

PCA highlighted a clear trend of the NW samples to cluster on the left side of PC1 (Figure 3A), which is characterized by lower loadings of all minerals and a higher cortisol concentration (Figure 3B). The negative relationship between cortisol and some mineral concentrations is confirmed by correlation analysis. In fact, cortisol was negatively correlated with Li (Rho = −0.548; *p* = 0.001), Co (Rho = −0.466; *p* < 0.01), As (Rho = −0.471; *p* < 0.01), Cd (Rho = −0.600; *p* < 0.01), Cr (Rho = −0.359; *p* < 0.05), and TI (Rho = −0.397; *p* < 0.05).

Descriptive statistics and results of the Mann–Whitney test for cortisol and mineral hair concentrations in each geographic area are reported in Table 1. The presence of significant differences in mineral concentration between culling areas was confirmed for some heavy metals (Al, As, Co, Cu, Cd and Ni). Differences in Cr and in cortisol concentrations approached statistical significance (*p* < 0.10).

Descriptive statistics and t-test results for biometrical measures and nutritional index in each geographic area are reported in Table 2. No significant differences between culling areas were recorded in terms of biometrical measures and nutritional index (KFI), except for a lower height at withers in calves from the SE area (SE: 90.86 ± 1.10 cm; *n* = 7; NW: 94.56 ± 1.22 cm; *n* = 8; *p* < 0.05).

## 4. Discussion

Minerals and cortisol are often correlated with each other and both of them are fundamental in many bodily functions, therefore their combined study is of scientific relevance.

A study on red deer in another Alpine area showed lower concentrations of Fe, Cu, Zn, Se, Cr, Ni, Cd and Pb [55] compared with our results. Hair mineral concentrations were also studied in other species; compared to this study, similar hair Zn and Fe concentrations were recorded in the American white-tailed roe deer [19], akin levels of Cu, Zn, and Fe were found in the Californian mule deer [15], and lower Fe, Zn and Cu in the Alaskan moose [14].

In all the above mentioned studies [14,15,19,55], and also in the present one, a high level of data dispersion was observed for most of the minerals, showing a great variability among samples, which is probably due to several different reasons (e.g., environment, analytical methods, etc.). Therefore, further studies based on a larger sample size would be advisable to allow the drawing of reliable conclusions. However, we can speculate some hypotheses, based on our results. First of all, some differences were recorded between the culling areas. Some of the minerals analyzed in this study were more concentrated in the SE area. These differences are clearly highlighted by the PCA and Spearman test, although univariate analysis could confirm the presence of statistical differences only for five minerals (Al, As, Ni, Co, Cu and Cd), with higher mineral concentrations in the SE area.

Regarding As results, a correspondence between water and hair analysis was recorded: in fact, higher As concentrations were recorded in animals coming from the SE area, where As concentrations in the water were actually beyond acceptable thresholds [66,70]. It is therefore possible to suppose a direct transfer of this mineral from the environment to hair, due to an important water contamination that allows rapid accumulation in animals drinking tainted water and eating on contaminated pastures. As mentioned before, hair concentrations in animals culled in the SE area were higher not only for As, but also for all other heavy minerals (Al, Co, Cu, Cd, V, Cr, Fe, Ni, Zn, Pb), although statistical differences between the areas were recorded only for Al, As, Co, Cu, Cd and Ni. Unfortunately, in the absence of a water analysis for all the heavy metals, it is not possible to confirm a correlation, but it is possible to speculate a mechanism similar to the one hypothesized for As and, in turn, an environmental pollution.

The importance to detect As contamination in wild red deer, as well as the other above mentioned heavy metals, is related to the possibility of meat contamination. Recently, the consumption of meat from wild ungulates has been increasing in Italy and in other European countries because of its large availability, ensuing the general increase of wild ungulate populations and culling [20,73,74,75]. In order to ensure meat safety, the assessment of heavy metals and other environmental contaminants cannot be neglected, because of the possible risks to public health. The consumption of meat from wild culled animals can actually be a marginal, even if increasing, problem; nevertheless, also domestic animals for food production (milk and meat) grazing on contaminated areas could, in turn, be contaminated, becoming a further risk for public health [76]. In this light, hair analysis could be an easy and rapid screening method, as reported by several authors, which also underlined the connections between hair and other tissue concentrations [51,56]. 

The study of metal contamination can be important for the protection of human health. In fact, metals can escape control mechanisms such as binding to specified cell constituents, compartmentalization, homeostasis, malfunctioning of the cellular processes, oxidative deterioration and transport, and therefore have toxic and lethal effects [77,78,79,80,81,82,83,84].

The results of this study also showed that animals culled in the SE area have lower cortisol levels, and, in spite of our limited sample size, this difference approached statistical significance (Figure 3; Table 1). This leads us to hypothesize that red deer in the NW area are subjected to a higher allostatic loads that refers to the price the body pays for being forced to adapt to adverse psychosocial or physical situations, and it represents either the presence of too much stress or the inefficient operation of the stress hormone response system, which must be turned on and then turned off again after the stressful situation is over [28]. In fact, Caslini et al. [36] suggested that hair cortisol concentrations provide a good index of the long-term hypothalamic–pituitary–adrenal (HPA) axis activity and allostatic load in red deer, as proved by the fact that individuals with higher cortisol concentrations in the hair derived from areas with higher population density, higher anthropic disturbances, and harsher environmental conditions. In our study area, the environmental conditions are actually more favorable in the SE sector, where pasture quality is higher and the abundant presence of trees, such as *Pinus cembra* and *Picea abies*, provides good shelter for the animals who can therefore feel more protected [80]. This may explain the observed differences in cortisol level and suggests that red deer in the two areas are subjected to a different allostatic load. Then, the different allostatic load could be due to the different characteristics of the water or soil, different dynamics among deer populations or territory use. In spite of this, we could find almost no difference in the weight, size nor nutritional status between the two areas. The only difference was that calves are slightly taller in the SE that in the NW area: this suggests that the higher allostatic load in the NW area can negatively affect calf development. However, sample size within each age class is too low to allow the drawing of any reliable conclusions.

Finally, the negative relationship between cortisol and mineral hair concentrations also deserves some attention. This relationship may be explained by the fact that, in humans, prolonged stress has been hypothesized to negatively affect the body’s mineral status through a physiological pathway [81,82,83]. This mechanism could act to hinder adequate absorption, distribution or excretion of minerals, increasing the body’s need for minerals through changes in metabolism or redistributing the minerals to tissues with higher requirements, although these mechanisms need to be further explored [25,33,34,35]. Singh et al. [84] described a decrease in plasma mineral concentrations in response to stress, due to glucocorticoid stimulation for hepatic metallothionein synthesis that in turn induces the sequestration of minerals by the liver. However, as indicated by Roy et al. [85], mineral deficiencies may activate the HPA axis, causing glucocorticoid production, suggesting that the cortisol–mineral relationship may also operate in the reverse direction. Reduced Fe levels after stress have been explained by an increase in ferritin concentrations (an intracellular Fe storage protein), indicating a shift from circulating to stored iron [84]. In this context, hair Fe could be considered an excretion or storage pathway. On the other hand, animal studies indicated decreased iron absorption in relation to psychological stress, possibly through changed expression of iron transporters [86]. Therefore, the differences of hair mineral concentrations in the two areas may be due to more than one factor (allostatic load, mineral absorption, environment, reverse direction with cortisol) that act together to produce the final results, and unfortunately our field study did not allow us to separate each singular effect.

## 5. Conclusions

In conclusion, this preliminary study represents a step forward in the study of wildlife allostatic load. A negative relationship was found between hair mineral and cortisol concentrations. Both of these factors are part of complex mechanisms linking ecological change with impaired wildlife population health. Hair mineral and cortisol assessment could be an easy, rapid, and low cost screening method. However, to serve as a useful management tool for wildlife, factors influencing hair mineral and cortisol concentrations and the respective threshold values must be identified. Moreover, these factors could be used in environmental studies, to assess contaminant presence in wild species and to determine risks for humans; and not last, in public health programs, to estimate contamination risks linked to the consumption of meat from wild animals or to assess for the presence of contaminants in food producing animals.

## Figures and Tables

**Table 1:** Number of animals, means, standard errors (SEM), minimum (Min) and maximum (Max) values of cortisol and mineral hair concentrations in each geographic area of Stelvio National Park (South East, SE and North West, NW). Significant differences (Mann–Whitney test) are highlighted in bold.

		SE Area					NW Area					*p*-Value ^a^
*Variable*	*Unit*	*n*	*Mean*	*SEM*	*Min*	*Max*	*n*	*Mean*	*SEM*	*Min*	*Max*	
Cortisol	pg/mg	16	5.18	0.34	3.41	7.18	15	6.97	0.63	4.33	11.61	0.063
Li	μg/g	16	0.53	0.11	0.15	1.55	15	0.31	0.07	0.00	1.02	0.206
B	μg/g	8	0.87	0.18	0.26	1.78	5	1.88	0.87	0.11	4.16	0.770
Al	μg/g	15	242.98	41.38	89.30	726.14	15	137.62	20.69	11.39	286.42	0.026 *
Ti	μg/g	16	4.24	0.43	0.29	6.59	15	3.95	0.57	0.82	8.47	0.477
V	μg/g	16	0.75	0.16	0.14	1.88	15	0.39	0.07	0.03	1.01	0.220
Cr	μg/g	16	0.53	0.05	0.16	0.93	15	0.42	0.07	0.17	1.28	0.053
Fe	μg/g	16	222.68	25.48	44.22	410.66	15	216.58	50.95	59.98	764.89	0.220
Ni	μg/g	16	1.32	0.28	0.27	3.97	14	0.64	0.18	0.14	2.67	0.022 *
Co	μg/g	16	0.18	0.02	0.06	0.35	14	0.09	0.01	0.05	0.15	0.003 **
Cu	μg/g	16	5.03	0.29	2.94	7.40	15	4.07	0.19	2.55	5.25	0.007 **
Zn	μg/g	16	70.43	1.15	64.74	83.04	15	65.41	2.07	45.71	74.55	0.123
As	μg/g	16	1.39	0.17	0.44	2.88	15	0.72	0.10	0.18	1.56	0.003 **
Ag	μg/g	8	0.05	0.01	0.01	0.10	9	0.07	0.02	0.01	0.18	0.630
Cd	μg/g	7	2.32	0.42	0.60	3.50	12	0.34	0.12	0.00	1.00	0.001 **
Sn	μg/g	11	0.14	0.03	0.04	0.33	9	0.12	0.03	0.03	0.33	0.494
Sb	μg/g	14	0.03	0.01	0.01	0.09	10	0.07	0.02	0.01	0.21	0.639
Ba	μg/g	15	2.24	0.29	0.81	4.66	15	2.04	0.45	0.20	7.09	0.419
Tl	μg/g	16	1.99	0.67	0.00	6.59	15	1.31	0.56	0.00	7.58	0.874
Pb	μg/g	12	0.75	0.16	0.08	1.69	11	0.35	0.08	0.01	1.03	0.110

^a^ Significance levels: * *p* < 0.05; ** *p* < 0.01.

**Table 2:** Descriptive statistics (number of animals, means, SEM) of biometrical measures and nutritional index (KFI) in each geographic area (SE and NW). Significant differences (*t*-test) are highlighted in bold.

		SE Area			NW Area			
*Variable*	*Unit*	*n*	*Mean*	*SEM*	*n*	*Mean*	*SEM*	*p*-Value ^a^
**Calves**								
body length	cm	7	126.00	4.17	8	134.87	3.21	0.111
foot length	cm	7	42.86	0.68	8	44.81	0.72	0.073
height at withers	cm	7	90.86	1.10	8	94.56	1.22	0.044 *
jaw length	mm	6	207.14	1.94	8	206.22	3.96	0.856
carcass weight	kg	7	30.00	2.027	8	33.31	3.00	0.391
kidney fat index	KFI	6	24.24	6.66	8	22.37	3.38	0.792
**Yearlings**								
body length	cm	4	148.00	3.76	2	145.00	5.00	0.665
foot length	cm	4	48.12	1.23	2	47.75	0.75	0.853
height at withers	cm	4	104.50	4.11	2	105.50	1.50	0.880
jaw length	mm	4	243.10	6.37	2	225.20	9.70	0.187
carcass weight	kg	4	50.75	3.04	2	44.00	5.00	0.286
kidney fat index	KFI	4	35.94	9.55	2	23.26	6.66	0.442
**Adult**								
body length	cm	5	162.50	1.05	5	166.20	3.20	0.304
foot length	cm	5	49.70	0.55	5	49.30	0.51	0.606
height at withers	cm	5	108.20	0.80	5	106.20	2.08	0.396
jaw length	mm	5	268.73	4.17	5	262.11	3.15	0.241
carcass weight	kg	5	58.80	3.83	5	59.90	3.38	0.835
kidney fat index	KFI	5	34.20	7.42	5	59.10	15.30	0.181

^a^ Significance level: * *p* < 0.05.
